# Design and Screening of a Glial Cell-Specific, Cell Penetrating Peptide for Therapeutic Applications in Multiple Sclerosis

**DOI:** 10.1371/journal.pone.0045501

**Published:** 2012-09-25

**Authors:** Corey Heffernan, Huseyin Sumer, Gilles J. Guillemin, Ursula Manuelpillai, Paul J. Verma

**Affiliations:** 1 Reprogramming and Stem Cell Laboratory, Centre for Reproduction & Development, Monash Institute of Medical Research, Monash University, Clayton, Victoria, Australia; 2 Placental Stem Cell Laboratory, Centre for Reproduction & Development, Monash Institute of Medical Research, Monash University, Clayton, Victoria, Australia; 3 Department of Pharmacology, University of New South Wales, Sydney, New South Wales, Australia; 4 South Australian Research and Development Industry, Turretfield Research Centre, Rosedale, South Australia, Australia; Université Pierre et Marie Curie-Paris6, INSERM, CNRS, France

## Abstract

Multiple Sclerosis (MS) is an autoimmune, neurodegenerative disease of the central nervous system (CNS) characterized by demyelination through glial cell loss. Current and proposed therapeutic strategies to arrest demyelination and/or promote further remyelination include: (i) modulation of the host immune system; and/or (ii) transplantation of myelinating/stem or progenitor cells to the circulation or sites of injury. However, significant drawbacks are inherent with both approaches. Cell penetrating peptides (CPP) are short amino acid sequences with an intrinsic ability to translocate across plasma membranes, and theoretically represent an attractive vector for delivery of therapeutic peptides or nanoparticles to glia to promote cell survival or remyelination. The CPPs described to date are commonly non-selective in the cell types they transduce, limiting their therapeutic application *in vivo*. Here, we describe a theoretical framework for design of a novel CPP sequence that selectively transduces human glial cells (excluding non-glial cell types), and conduct preliminary screens of purified, recombinant CPPs with immature and matured human oligodendrocytes and astrocytes, and two non-glial cell types. A candidate peptide, termed TD2.2, consistently transduced glial cells, was significantly more effective at transducing immature oligodendrocytes than matured progeny, and was virtually incapable of transducing two non-glial cell types: (i) human neural cells and (ii) human dermal fibroblasts. Time-lapse confocal microscopy confirms trafficking of TD2.2 (fused to EGFP) to mature oligodendrocytes 3–6 hours after protein application *in vitro*. We propose selectivity of TD2.2 for glial cells represents a new therapeutic strategy for the treatment of glial-related disease, such as MS.

## Introduction

Multiple sclerosis (MS) is characterized by auto-immunological destruction of myelinating oligodendrocytes of the central nervous system (CNS), resulting in denuding of conducting neural cells, and focal plaques of inflammation. An array of endogenous and exogenous inducers of MS aetiology have been proposed, including deficiencies in oligodendrocyte precursor maturation, limiting remyelination of denuded axons, and/or proteolytic cleavage of oligodendrocyte-derived protein initiating axon-glial cell dissociation [1]. However, our current therapeutic arsenal is primarily limited to repression of host immune response with (i) recombinant interferons, or (ii) immunomodulatory and immunosuppressive agents that target immune cell activation, function or migration. The inter-patient variability in effectiveness of these agents [2], as well as secondary risks associated with immunological modulation, highlight the need for development of alternative therapeutic strategies for this debilitating disease. Design and delivery of specific (non-immunological) therapeutic proteins to the brain of MS sufferers has received little attention as an alternative treatment for MS, and conceptually circumvents the problems associated with proposed myelinating cell delivery.

The intrinsic ability of cell penetrating peptides (CPP) to translocate plasma membranes of target cells can be exploited to deliver therapeutic proteins/peptides to target cells (reviewed [3]). A number of non-selective CPP’s have been described and well characterized, modelled on neural development or observations of viral envelope protein/cell interactions during infection. For example, the Antennapaedia homeoprotein (Antp) in *Drosophila melanogaster* was originally shown to regulate neural development by translocating to the nuclear compartment of neurones to repress transcription [4]. Subsequently, an N-terminal, 15 amino acid peptide (RQIKIWFQNRRMKWK) [4], [5] within the third helix of the homeodomain of Antp was responsible for transmembrane transduction (plasma and nuclear membranes). From this sequence, a CPP named ‘Penetratin’ was devised and has since been extensively characterized [4]. Similarly, HIV transcriptional-activator of transcription (Tat) protein co-binds (i) viral envelope protein GP120 and (ii) host cell heparan sulfate proteoglycans to mediate transmembrane import (infection) through caveolar (‘lipid raft’) endocytosis [6]–[10]. From the full length protein, an arginine-rich, basic domain of Tat (^49^-RKKRRQRRR-^57^) undertakes rapid (in the order of minutes; [10]) and efficient translocation to the nuclear compartment of target cells, making Tat an ideal fusion partner for delivery of recombinant transcriptional activators to nuclear chromatin [11], [12]. Similarly, domains of 9–11 cationic amino acids (eg. polyarginine, RRRRRRRRR) are also effective vehicles for non-selective cytosolic/nuclear delivery of functional proteins/domains [13], [14]. The positive charge of arginine, and the ability of it guanidinium side chains to form hydrogen bonds with sulfate or phosphate groups of cell surface proteins, are both proposed to contribute to its association with plasma membrane and subsequent translocation capability. However, the promiscuous nature of their target cell range limits their therapeutic relevance.

Tetanus toxin fragment-C (TTC) selectively binds ganglioside GT1b on lower, and spinal cord motor neurons before internalization and even retrograde trans-synaptic transport [15]. Through fusion to TTC, a number of functional, therapeutic proteins have been delivered to motor neurons in a cell-specific manner *in vivo* for proof-of-concept treatment of amyloid lateral sclerosis (ALS) and Parkinson’s Disease, such as human insulin-like growth factor-1 (hIGF-1; [15]), human Cu/Zn superoxide dismutase (hSOD-1; [16]) and glial cell line derived neurotrophic factor (GDNF; [17]). To our knowledge, a human glial cell-specific CPP has not been described to date.

A number of infections manifest in human glial cells, with viral:host protein:protein interactions conceptually forming a theoretical framework to which a glial cell-specific targeting peptide could be devised, in a similar way to Tat and TTC peptides [4]–[12], [15]–[17]. For example, reactivation of JC (John Cunningham) virus in infected human glial cells, usually upon acquisition of compromised immune system, causes onset of a demyelinating disease called *Progressive multifocal leukoencephalopathy* (PML). However, JC viral-specific markers have also been detected in a range of other tissues and cells, including cerebrospinal fluid and urine, tonsil and renal cells, bone marrow and circulating lymphocytes, lungs and gastrointestinal tract (reviewed [18]). Lymphocytic Choriomenigitis Virus (LCMV) is an Old World arenavirus detected in meningeal membranes of the brain as well as glia (although, particular viral variants also infect CD11c^+^ dendritic cells *in vitro* or spleen; [19]), whereby ‘GP1’ viral surface glycoproteins bind the extracellular portion of the glial cell-derived protein, a-dystroglycan (a-D; [19], [20]).

Glial cells primarily comprise astrocytes and oligodendrocytes. Astrocytes provide structural support for cranial interstitial cells, and proliferate in response to inflammation to lay down scar tissue and secrete factors that inhibit oligodendrocyte progenitor maturation [21], [22]. In the ventral aspect of the developing spinal cord, activation of *Oligodendrocyte lineage gene 2* (Olig2) directs specification of oligodendrocyte precursors (and motor neurons) from a common precursor pool [23]. We aimed to devise a peptide sequence that selectively binds and transports across glial cell plasma membranes, and hypothesized a glial cell-specific CPP could be modelled on protein-protein interactions between glia and a glia-(semi)selective virus, such as LCMV.

## Methods

### Materials

Reagents were purchased from Sigma-Aldrich (Castle Hill, NSW, Australia) unless otherwise stated. All DNA sequencing was performed at the Gandel Sequencing Facility, Monash Institute of Medical Research, Australia.

### Methods

#### Theoretical Framework for Design of a Putative Human Glial Cell-Specific Targeting Peptide

LCMV is an Old World arenavirus that (semi-selectively) infects glial cells of the CNS through interactions with (i) its own surface protein ‘GP1’ and (ii) the extracellular portion of the glial cell-derived protein, alpha-dystroglycan (a-D; [20]). In non-infected individuals, glial-derived a-D commonly binds numerous extracellular matrix proteins with high affinity, including the neuromuscular proteoglycan *agrin* and basal lamina proteins *laminin-1/-2* of the peripheral nervous system [24]–[37]. Although the a-D residues implicated in binding LCMV GP1 have been described [19], the a-D-interacting motifs of GP1 remain unknown. To elucidate the (semi-selective) glial-derived, a-D-binding domain/s of GP1, thus representing a putative glial-specific CPP, we hypothesized protein sequence alignments of GP1 and other a-D-binding proteins (eg. laminin-2, agrin) may highlight domains of GP1 that undertake the interaction with a-D.

#### Culture of Cell Lines

Since immortalized lines of mature human oligodendrocytes are lacking, we utilized a well-characterised oligodendrocyte precursor cell line (‘MO3.13’) [28]; human rhabdomyosarcomaRD:adult oligodendrocyte hybrid; [29], [30]). Incubation of MO3.13 with the protein kinase C activator 4-beta-phorbol 12-myristate (PMA) with serum starvation for 5 days initiated differentiation to mature oligodendrocytes of characteristic spindle-shaped morphology and limited proliferative potential [29](G.J. Guillemin, personal communication; Figure S1A). Human foetal astrocytes (2nd trimester aborted foetus) were matured to non-proliferative derivatives by 5 days of serum starvation (U.Manuelpillai, personal communication). Human oligodendrocyte and astrocyte precursor cells (and their matured progeny), as well as human dermal fibroblasts (‘Detroit 551’ line; American Type Culture Collection [ATCC], USA) were cultured in DMEM (Invitrogen, Australia), 10% (v/v) foetal bovine serum (FBS; JRH, Australia) and 0.5% (v/v) penicillin/streptomycin. Human neural stem cells were cultured via manufacturer’s instructions (Gibco Human Neural Stem Cells [H9-Derived]; Life Technologies, Australia).

#### Construction of CPP Protein Expression Vectors: Histidine-tagged “TD1, TD2, TD1.1, TD2.1 and TD2.2” Constructs

**Figure 1:**
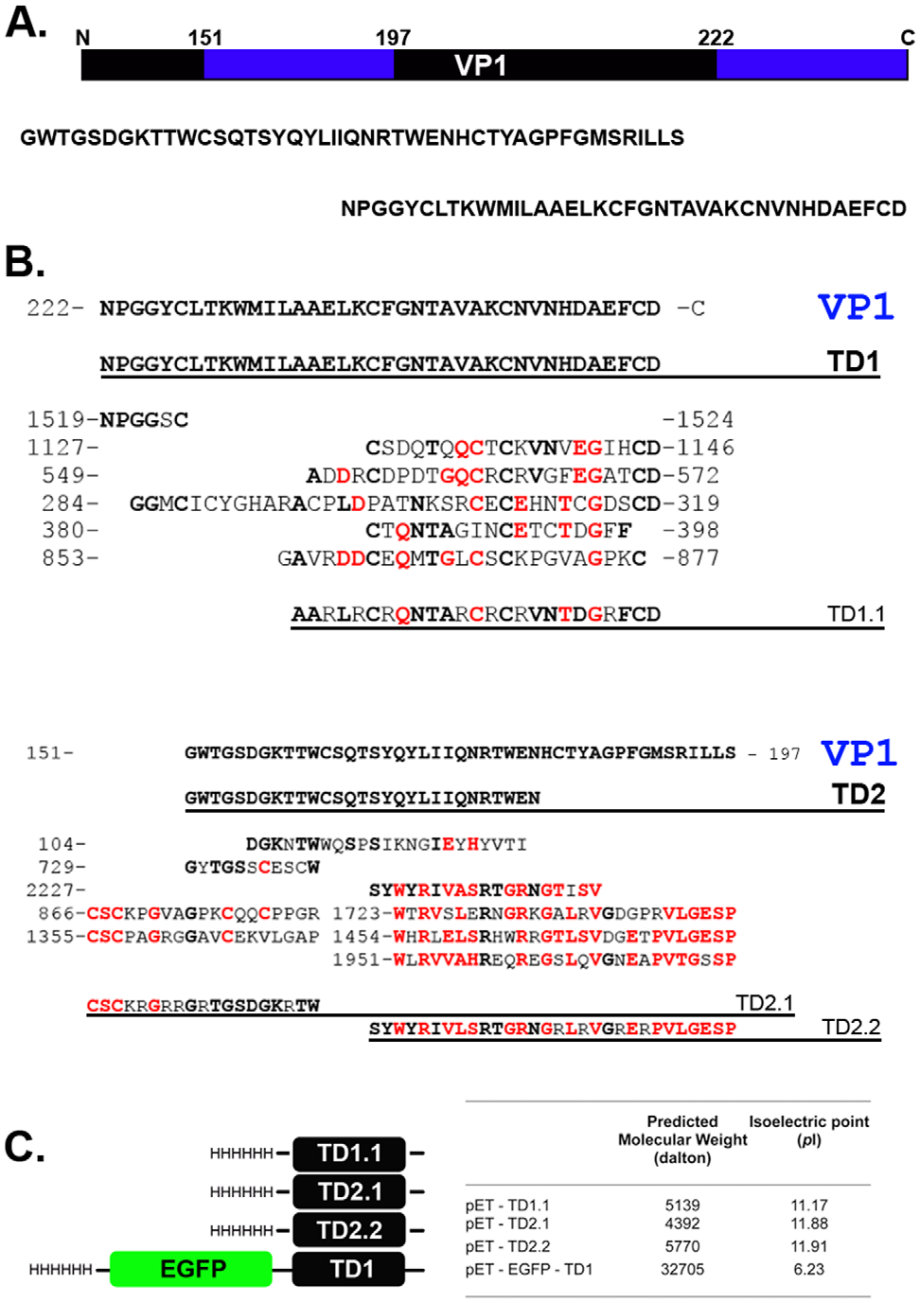
Theoretical Design of Glial-specific Targeting Peptides. (A) Illustration of full-length viral glycoprotein-1 (GP1) of Lymphocytic Choriomeningitis Virus (LCMV) to which Transduction Domains (TD)-1, TD-2, TD-1.1, TD-2.1 & TD-2.2 were devised. Blue bars represent domains within the amino acid sequence of GP1 that are conserved with other a-dystroglycan binding proteins (Laminin2 and Agrin; see B for protein sequence alignments). TD’s are devised from these conserved regions. (B) Protein sequence alignments of GP1 and Laminin-2 or Agrin. Amino acid domains of Laminin-2 or Agrin are denoted and numbered to each side. TD1 and TD2 are identical to GP1 domains. TD1.1, TD2.1 & TD2.2 are composite sequences of GP1/Laminin-2 and Agrin; residues were included if any of the Laminin-2 or Agrin conserved sequences had identical amino acids to the reference GP1 sequence (shown in black). Where identical residues could not be found, amino acids represented more than once among the Laminin-2 and Agrin sequences were included (shown in red). Where no similarity was observed between reference GP1 and Laminin-2/Agrin sequences, cationic residues (R, K) were included for their transmembrane transduction abilities (in normal type; reviewed [35]). (C) Diagrammatic outline of the transduction domain constructs. TD1.1, TD-2.1 & TD-2.2 are fused to a histidine leader sequence. TD1 was fused to EGFP (interspaced by a flexible -SGGGS- linker sequence) and a histidine leader sequence. Right are predicted molecular weights (Dalton) and isoelectric points (pI) of pET-TD proteins (MW & pI both ascertained with ExPasy software; http://ca.expasy.org/tools/pi_tool.html).

**Figure 2:**
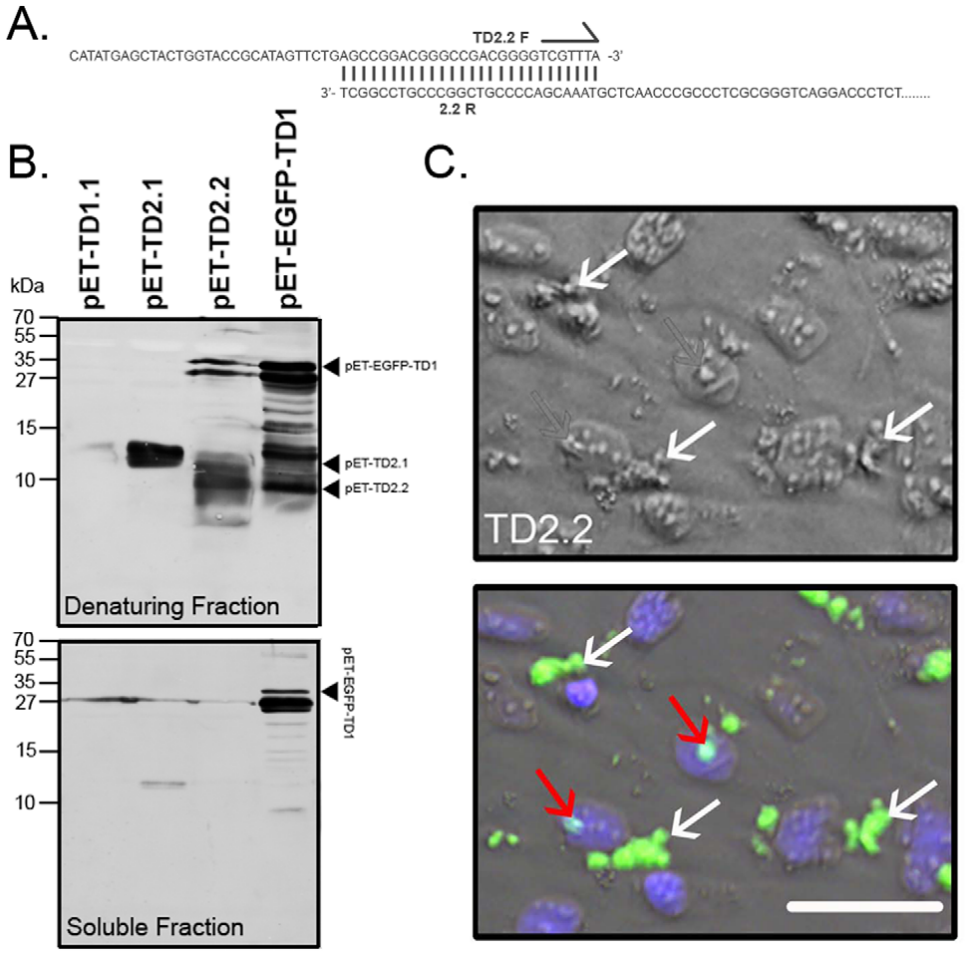
Construction and Optimization of TD domain Purification, and Initial *in vitro* Screening. (A) Representation of overlapping PCR primers for generation of novel His-tagged PCR products for cloning to protein expression vectors. (B) Western Blot of expressed pET-Transduction domain (TD) recombinant proteins purified under insoluble (denaturing) conditions (top blot) and soluble (bottom blot) conditions. Predicted proteins are indicated to right of each gel. (C) Oligodendrocyte cultures treated with TD2.2 recombinant protein showing TD2.2 localized to apparent cytosolic (white arrows) and nuclear (red arrows) compartments. Scale bars: 50 µM.

For short, novel ‘TD’ PCR products (for figures 1 & 2), reverse-complimenting (overlapping) oligonucleotides were devised incorporating the common histidine-tag and each CPP sequence (termed ‘TD’) interspersed with a flexible linker (-SGGGS-) linker for amplification in PCR lacking template. During PCR, annealed oligonucleotides extended to form the CPP inserts (example outlined Figure 2A). PCR products were excised from agarose gel, cloned to pGEM cloning vectors (Promega, Australia) before clones with insert (blue/white colony selection) underwent DNA sequencing (Figure S2A). Following restriction endonuclease digestion from cloning vectors of confirmed sequence, DNA inserts were ligated to digested pET protein expression vectors (Novagen/Merck, Australia), electro-transformed to BL21(DE3) *E. coli* (Stratagene, USA) and plated to agar plates (prepared in-house). The following day, clones were screened for the presence of insert (by PCR) and positive clones sequenced. Glycerol stocks of successful clonal constructs were kept at −80°C. Histidine-tagged TD1, TD2, TD1.1, TD2.1 and TD2.2 recombinant proteins were induced and purified as outlined below.

#### Histidine-tagged EGFP-TD2.2 Construct

**Figure 3:**
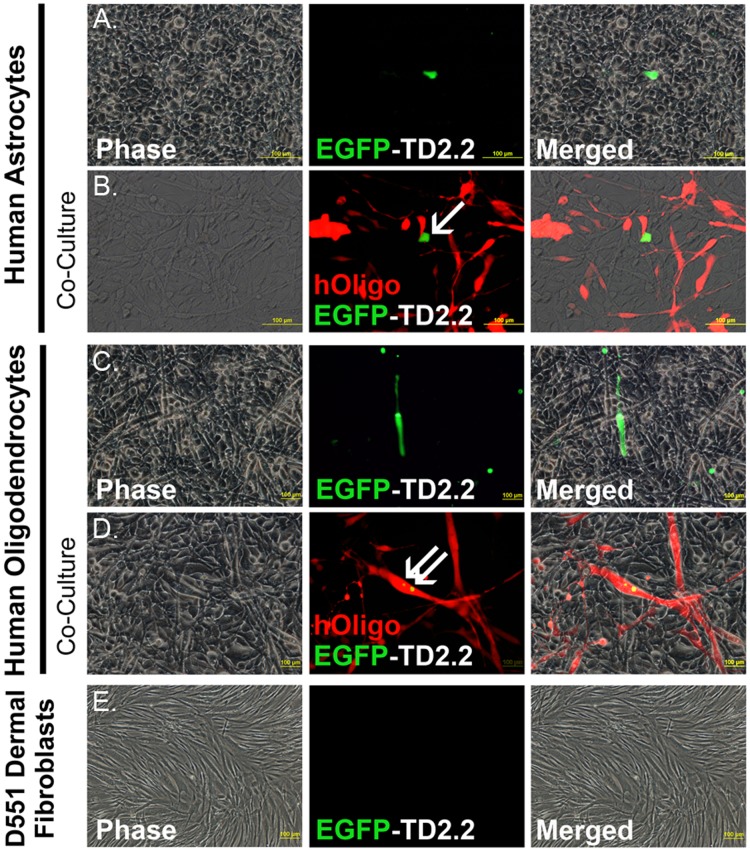
Representative (n = 3) Immunofluorescence Imaging of EGFP-TD2.2 Delivery Experiments. Rows A, C & E show phase (left panels), combined fluorescence (middle panels) and merged images (right panels) of mature human astrocytes, mature human oligodendrocytes and human dermal fibroblasts, respectively. Rows B & D show co-cultured of human astrocytes (unlabelled) and mCherry^+^ human oligodendrocytes. Arrows highlight respective cell types transduced EGFP-TD2.2 recombinant protein.

**Figure 4:**
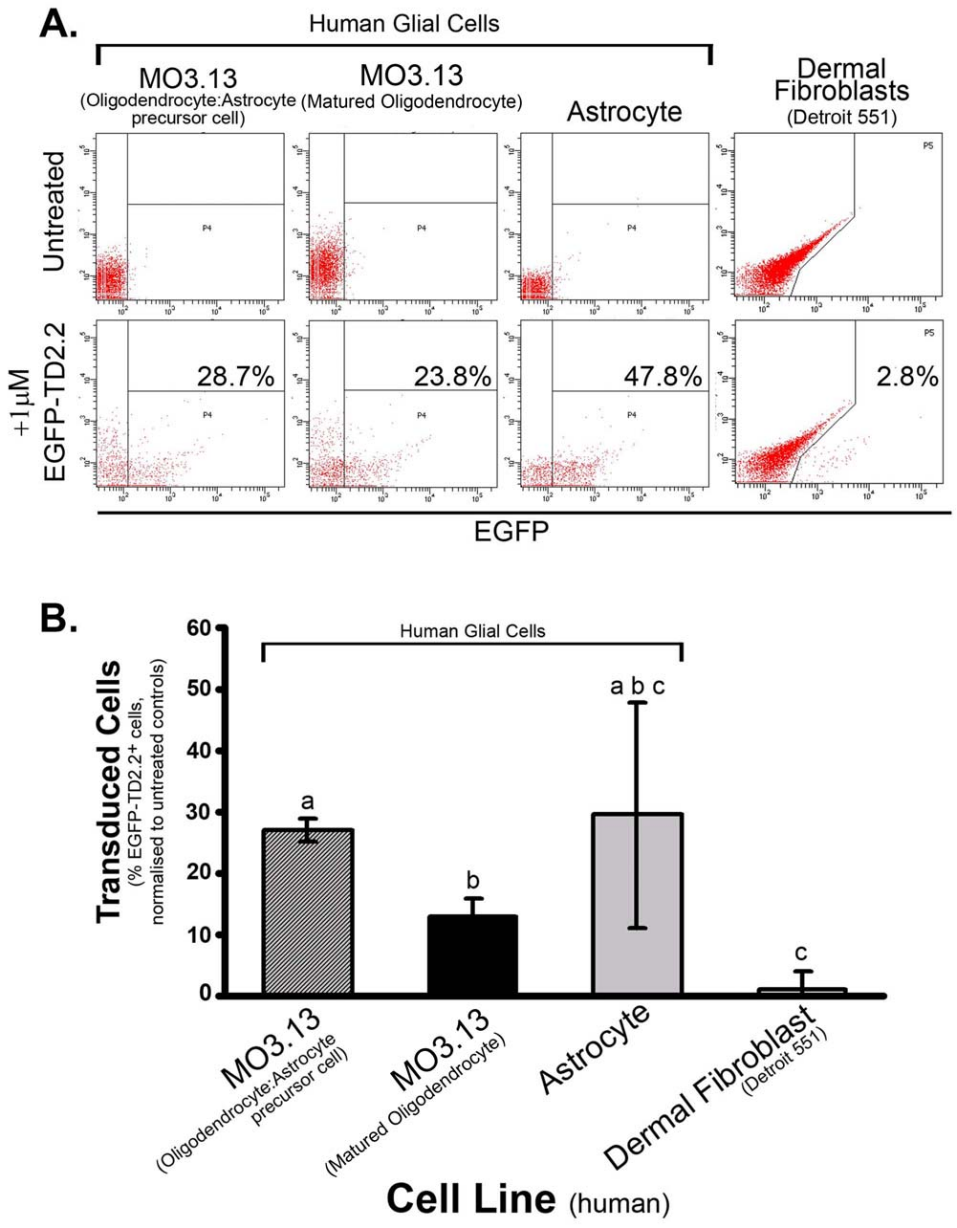
Representative (n = 3) Flow Cytometry Analysis of Relative Proportions of Human Astrocytes, Immature/Mature Oligodendrocytes & Dermal Fibroblasts to undertake EGFP-TD2.2 Transduction. (A) Representative Flow Cytometry plots of each cell type. Top panels show untreated cells from which gated events were devised for treated cells (bottom panels). X axis is EGFP; EGFP^+^ events denotes as a percentage of total event for each plot (bottom). (B) Combined statistics of EGFP-TD2.2 delivery experiments. T-tests of combined mean (±SEM) EGFP-TD2.2^+^ events across n = 3 experiments defined significance. Groups with the same superscript denotes non significant difference.

**Figure 5:**
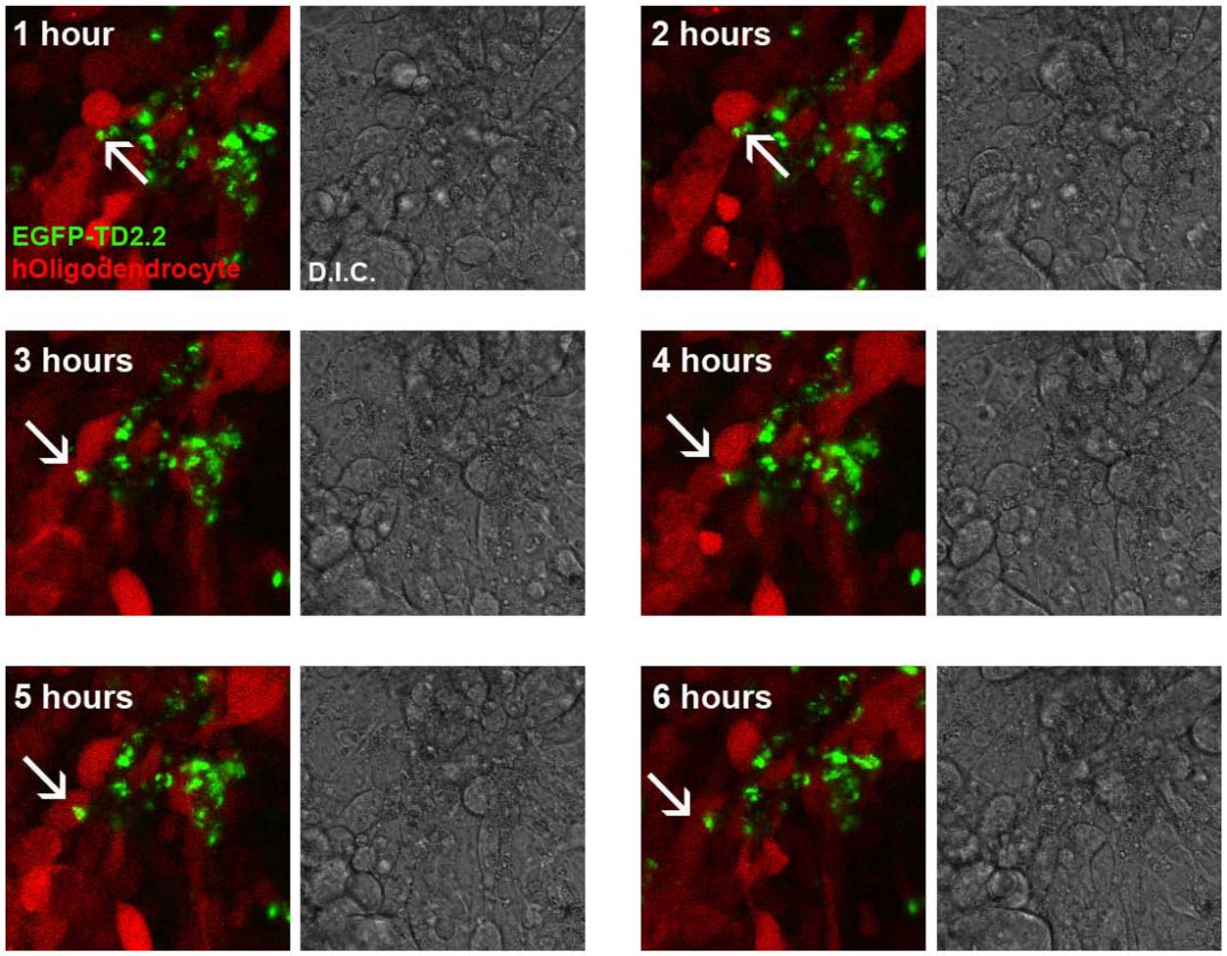
Kinetics of EGFP-TD2.2 Recombinant Protein Transduction to Mature Human Oligodendrocytes by Sectional Confocal Microscopy. A single field of view (single mid-sectional depth) of mCherry^+^ human oligodendrocytes, with hourly differential interference contract (DIC) and mCherry/EGFP images taken following application of 1 µM EGFP-TD2.2 recombinant protein. Arrows track transduction of extracellular EGFP-TD2.2 protein into an oligodendrocyte.

To construct the pET-EGFP-TD2.2 protein expression vector (for Figures 3, 4 and 5), DNA inserts of EGFP and TD2.2 were amplified by PCR with complimentary *NcoI* (N-terminal), *BsrGI* (middle) and *XhoI* (C-terminal) restriction endonuclease digest sites (NEB Biolabs, Australia; Figure S3). DNA inserts were ligated to pGEM-T cloning vectors before DNA sequencing. Clones with confirmed inserts underwent subsequent digestion before dual ligation to pET28-c (Figure S3). Transformation to BL21 (DE3) *E.coli* generated clones that were screened (by restriction digest) for the presence of the DNA insert before DNA sequencing. EGFP-TD2.2 protein expression was induced as outlined below.

#### Expression, Purification and Concentration of all Histidine-tagged Recombinant Proteins

Expression of TD and EGFP-TD2.2 recombinant protein was performed through expansion in 200 mL LB Broth (made in-house) in the presence of 0.1 mM isopropyl beta–D-thiogalactosidase (IPTG; 230 rpm agitation overnight, 30–37°C). Initially, soluble and insoluble (requiring purification of denatured protein) fractions of expressed TD and EGFP-TD2.2 protein were purified by ion affinity chromatography (Ni-NTA columns; Qiagen, Australia) before Western Blot was performed (Figure 2B, Figure S3).

For purification of soluble recombinant proteins, bacteria were lysed (1% Triton X-100, 0.1 mg/ml Lysozyme in 50 mM NaH_2_PO_4_, 300 mM NaCl, 10 mM imidazole, pH 8.0) and incubated at 37°C for 1 hour, and on ice for 30 min. RNase (5 µg/ml; Invitrogen, Australia), DNase (2 units/ml; Promega, Australia) in 1 mM MgCl_2_ and phenylmethylsulfonyl fluoride (1 mM) were added before sonication on ice. Cells were further homogenized through a 23-gauge needle before centrifugation (9000×*g*, 30 min, 4°C). The supernatant was collected for purification/elution through Ni-NTA columns via manufacturer’s instructions. For purification of insoluble proteins, bacteria underwent urea denaturation following manufacturers instructions (Ni-NTA; Qiagen, Australia).

To concentrate protein preparations, four parts ice-cold acetone was added to one part purified protein (v/v) and incubated at −20°C for 30–45 min. Following centrifugation at 6800×g for 10 min (4°C), proteins pellets were resuspended in 50 µl sterile PBS. Molar concentrations of proteins were calculated by protein spectrophotometry.

#### Application, and Immunocytochemical Detection, of Transduction Domain (TD) Recombinant Proteins to cells *in vitro*

Matured human oligodendrocytes were plated to duplicate culture plates (approximately 1×10^5^ per cm^2^) and incubated overnight. The following day, culture media was changed to DMEM only and purified pET-EGFP-TD1, pET-TD1.1, pET-TD2.1 and pET-TD2.2 (ranging 15–49 µg/200 µL DMEM) recombinant proteins applied to duplicate cultures of oligodendrocytes for 3 hours (top panels, Figure S2B). One duplicate well (“pre-acid wash”) was fixed in paraformaldehyde, cell membranes permeabilized with 0.05% (v/v) Triton-X100 before recombinant protein was detected with antibody recognizing the histidine leader sequence (Abcam/Sapphire, Australia; top panels; Figure S2B). Cells of the second duplicate underwent 4 washes to remove peripherally bound, non-transduced recombinant proteins; 3×PBS and 1×brief (1.5 min) wash in 0.2 M Glycine-HCl (prepared in-house; [31]) before fixation, permeabilization and antibody labelling as outlined above (bottom panels; Figure S2B). Cells treated with pET-EGFP-TD1 were imaged (for EGFP detection) before and after acid wash (i.e. no antibody labelling).

For EGFP-TD2.2 delivery experiments (Figures 3, 4 and 5), human astrocytes, human dermal fibroblasts and matured mCherry^+^ human oligodendrocytes were plated in isolation at a total cell density approximately 1×10^5^ cells/cm^2^ (rows A, E, C respectively, Figure 3). Immature, mCherry^+^ human MO3.13 precursor oligodendrocytes, and human neural cells, were also plated at the same density (not shown). To mimic the cellular environment in the brain, human astrocytes and matured mCherry^+^ human MO3.13 oligodendrocytes were plated at a ratio of 5∶1 (rows B & D, Figure 3; same total cell density). The following day, cells were washed and culture media changed to DMEM only before 1 µM purified EGFP-TD2.2 recombinant protein was applied to all cells. After 12 hours, cells were washed and 1 µM purified EGFP-TD2.2 recombinant protein reapplied (three administrations over 36 hours total). After the final 12 hour incubation, cells were washed in PBS before 0.2 M Glycine-HCl to remove surface-bound (ie. non-transduced) protein before transduction of EGFP-TD2.2 confirmed (i) visually, by immunofluorescent imaging, and (ii) percentage transduced cells in each individual cell population ascertained by flow cytometry analysis (Figures 3 & 4, respectively). Images in Figure 3 and 4A are representative of 3 independent experiments.

#### Western Blot

Western Blot was performed as described [32]. Briefly, 40 µg of reduced and heat-denatured protein for each TD preparation (both soluble and denaturing/insoluble conditions) were electrophoresed through 12–20% sodium dodecyl sulfate (SDS)- polyacrylimide protein gels (prepared in-house) before transfer to polyvinylidene difluoride (PVDF; Millipore, Australia) membranes. Blotted wet membrane was blocked with Odyssey blocking Buffer (Odyssey, Australia) and probed with anti-6x His-tag primary antibody (1∶2500; Sapphire/Abcam, Australia). Primary antibody detection was performed with Alexa Fluor, anti-mouse 680 secondary antibody (Molecular Probes/Invitrogen, Australia) for 1 h at room temperature. Test and negative control membranes (secondary antibody only) were visualized on an Odyssey InfraRed Imager (LI-COR Biosciences, Lincoln, NE, USA; intensity: 3–10, quality: medium, resolution: 169). For reference, predicted molecular weight of each TD recombinant protein was ascertained using ExPasy software (http://ca.expasy.org/tools/pi_tool.html; Figure 1C).

#### Flow Cytometry (FACS)

EGFP-TD2.2 treated cells were detached from the culture dish with Trypsin/EDTA (before the enzyme was deactivated with serum-containing culture media) and suspension centrifuged to pellet cells. The cell pellet was washed twice in ice-cold PBS (without CaCl_2_/MgCl_2_) before FACS performed on a Becton Dickinson FACS Canto II flow cytometer with BD FACS Diva software (V6.0). At least 5,000 unicellular events were analysed for each experimental condition.

#### Retroviral Transduction of Immature MO3.13 Human Oligodendrocytes with (CMV)-Cherry Transgene

The pRH(CMV) L-mCherry-myc retroviral vector was a generous gift from Kathryn Hjerrild, MIMR. Platinum-A cells (generation of amphotropic retrovirus) were grown in ‘FP’ medium containing 1 mg/ml puromycin and 10 mg/ml of blastcidin-S (antibiotic selection for expression of both viral envelope and key enzyme genes, env and gag-pol respectively; [33]) before plating at a density of 8×10^6^ cells/100 cm^2^. The following day, 27 µL of Fugene-6 transfection reagent (brought to room temperature; Roche Australia) was added to 0.3 ml of DMEM in a 1.5-ml Eppendorf tube (for each transgene) and mixed gently by finger tapping. After 5 min incubation (RT), 9 µg of pRH(CMV) L-mCherry-myc plasmid DNA was added drop-wise into the Fugene-6/DMEM-containing tube, mixed gently by finger tapping and incubated for 15 min. The transfection mixture was added drop-wise to the Plat-E/Plat-A dish and incubated overnight at 37°C. Media on Platinum-A-transfected plates was replaced with fresh FP medium (without antibiotics) the following morning, and cell cultures returned to the incubator overnight. Target cells were plated (8×10^5^ per 100 cm^2^ oligodendrocyte). The following day, media-containing retrovirus was filtering through a 0.45 mm cellulose acetate filter, polybrene solution (hexamethadine bromide; final 4 mg/ml) was added for neutralization of target cell surface charge and applied to cultures of target cells. Two consecutive retroviral infections of target cells took place overnight at 37°C, after which media was replaced with fresh culture media the following day. Infected oligodendrocytes were sorted for high *mCherry* expression (Figure S4A) and expanded in culture (Figure S4B).

#### Kinetics of pET-EGFP-TD2.2 Recombinant Protein Transduction to Mature Human Oligodendrocytes by Sectional Confocal Microscopy

1.25×10^5^ mCherry^+^ oligodendrocytes were plated to 8 well slides (1 µL volume, suitable for confocal microscopy; Ibidi GmBH, Germany). 1 µM pET-EGFP-TD2.2 was applied to cells immediately before the chamber slide was transferred to a heated (37°C) and humidified chamber on a confocal microscope (Leica, Australia). Four fields of view and mid-sectional depth were nominated, at which individual (i) differential interference contract (DIC), (ii) mCherry and (iii) (E)GFP images taken every hour for 48 hours (hourly images <6 hours shown Figure 5). Using mCherry expression/localization at mid-sectional plane as an indication of the total volume of oligodendrocytes, sectional imaging enabled tracking of the temporal kinetics of EGFP-TD2.2 transduction to human oligodendrocytes *in vitro*.

#### Statistical Analysis

T-tests of combined mean (±SEM) EGFP-TD2.2^+^ flow cytometric events across n = 3 experiments defined significance (p<0.05; Figure 4B). Groups with the same superscript denotes non significant difference.

## Results

### Design of Putative Glial Cell-specific Targeting Peptides

Firstly, to confirm MO3.13 oligodendrocytes express a-D, RT-PCR was performed on cDNA generated from these cells. Endogenous dystroglycan precursor transcript was detected in PMA-matured oligodendrocytes (Figure S1B; note, since a- and β-dystroglycan are alternatively spliced from the same transcript, primers amplified a 485 bp product of the precursor transcript for both human a- and β-dystroglycan; [34]). In human astrocytes, a faint PCR product was amplified, implying (i) a minor contaminating population of oligodendrocytes, or (ii) low expression of the precursor transcript for human a- and β-dystroglycan [34].

Amino acid sequence alignments of full-length LCMV GP1 (RefSEQ Accession# NP_694851), human laminin-2 (RefSEQ Accession# NP_000417) and human agrin (RefSEQ Accession# NP_940978) highlighted two domains of GP1 (amino acids 151–197 and 222-C terminus; Figure 1A) that ranged in 4%–45% identity to a number of domains of laminin-2 and agrin (identity shown as bolded black residues, Figure 1 A&B). Furthermore, the same laminin-2 and agrin domains showed greater conservation between themselves (ranging 9%–68% identity; identity shown as red residues in figure 1B). From these alignments, transduction domains termed ‘TD1’ and ‘TD2’ were devised and constructed identical to the two conserved regions of GP1. Additional transduction domains ‘TD1.1’, ‘TD2.1’ and ‘TD2.2’, comprising a composite of GP1-laminin2/agrin, and laminin2/agrin only; residues were included if they were represented in GP1 and/either laminin-2 and agrin domains (bolded black residues, figure 1B). If residues were conserved between laminin-2 and agrin only, they were also included (bolded red residues, figure 1B). When no common amino acids were found between GP1, laminin-2 or agrin for a particular residue, the cationic residue arginine (R) was inserted for its membrane transduction properties (reviewed [35]; Figure 1B). For these composite sequences, it was hypothesized conserved residues would impart (glial) specificity, whilst R would enhance plasma membrane transduction [11], [12]. Therefore, protein expression vectors were constructed for each of the five putative TD’s (shown in figure 2A and outlined in methods; note: despite five attempts, TD2 failed to clone and thus was omitted from the study).

To ensure recombinant protein of predicted molecular weight (MW) was expressed and purified, predicted MW and isoelectric points (*pI*) of each protein were established using EXpasy software (http://ca.expasy.org/tools/pi_tool.html; Figure 1C). pET-TD1.1, pET-TD2.1 and pET-TD2.2 all have *pI* (ie. pH at which the peptide is of neutral charge) of 11.1–11.9 (tabled figure 1C). In solution pH<11 (eg. culture media at 37°C/5%CO_2_/5%N_2_), these peptides are positively charged. Thus, the charge-attraction of the positively charged TD’s and the (weakly) negatively charged, resting potential of the cellular plasma membrane was hypothesized to promote their initial interaction before transduction to the cytosol.

### Construction & Purification of Numerous Putative Glial Cell-specific Targeting Peptides

The solubility of recombinant proteins can vary, with proteins enriched in nonpolar amino acids being relatively insoluble and thus requiring purification from inclusion bodies. To confirm the solubility of each TD protein, thus dictating the optimal purification method, (i.e. soluble protein directly from bacterial cytoplast, or through urea denaturation of inclusion bodies), recombinant protein expression was induced for each TD clone and purification performed under both soluble and denaturing (insoluble) conditions. Western blot was performed on each preparation (Figure 2B) against predicted MW of each recombinant protein outlined in figure 1C. Purified TD1.1 recombinant protein was not detected at predicted molecular weight of 5.139 kDa in either protein fraction (Figure 2B). A band of approximately 27 kDa was observed in the soluble protein fraction, but appears to be a contaminating band in a number of the soluble TD preparations. TD2.1 of predicted MW (4.392 kDa) failed to express and/or purify in either fraction either, however a considerable population of insoluble protein of approximately 12 kDa was purified and detected. It is unlikely this is a contaminating band from bacteria, as it is not represented in other samples (Figure 2B). Although the protein preparations were heat-denatured before loading on the gel, this band may still represent a TD2.1 homodimer, or heterodimer with unidentified, contaminating bacterial protein/s. A primary band of approximate, predicted MW (5.77 kDa) for TD2.2 was observed when purified under denaturing conditions only (Figure 2B). EGFP-TD1 purified under both soluble and insoluble condition, but considerably more protein was purified under denaturing conditions. Therefore, EGFP-TD1, TD2.1 and TD2.2 were purified under denaturing conditions for future experiments.

### Initial Screening of Prospective Glial Cell-specific Targeting Peptides for Transduction to Human Oligodendrocytes

To screen the transduction capacity of TD recombinant protein, purified EGFP-TD1, TD2.1 and TD2.2 (ranging 15–49 µg/200 µL) recombinant proteins were applied to duplicate cultures of matured oligodendrocytes for 3 hours (top panels, Figure S2B). One duplicate (“pre-acid wash”) was fixed, cell membranes permeabilized and recombinant protein detected with antibody recognizing the histidine leader sequence. To remove peripherally-bound (i.e. non-transduced) recombinant proteins from the other duplicate (“post-acid wash”), mature oligodendrocytes underwent an acid wash before fixation [31], permeabilization and antibody labelling as outlined above (bottom panels; Figure S2B). Cells treated with pET-EGFP-TD1 were imaged (for EGFP detection) before and after acid wash (i.e. no antibody labelling). Detection of TD1.1 and TD2.1 recombinant protein in pre-washed, oligodendrocyte cultures was sparse, and totally lost after acid washing (Figure S2B, compare top and bottom panels, left panels, TD2.1 representative of TD1.1). Despite being almost completely lost, one oligodendrocyte retained considerable TD2.1 protein (see white arrow, Figure S2B). In contrast, granules of TD2.2 were detectable after the acid wash, spatially localized to both nuclear (red arrows, figure 2C) and cytosolic compartments, but enriched in the cytosol (white arrows, figure 2C).

### Delivery of EGFP-TD2.2 Recombinant Protein to Human Glial Cells in vitro: Construction of the pET-EGFP-TD2.2 Protein Expression Vector

A protein expression vector was constructed incorporating EGFP and TD2.2 sequences, intersected by a flexible linker (outlined Figure S3). Predicted MW and *pI* of EGFP-TD2.2 recombinant protein was 32.18 kDa and 6.95 respectively (ExPasy software). Western Blot analysis of expressed EGFP-TD2.2 recombinant protein, detected with the aid of primary antibody recognizing the histidine tag, confirmed presence of recombinant protein of predicted molecular weight in both soluble and denaturing protein fractions (Figure S3). Additional protein populations were detected when protein was purified under denaturing conditions (bands above and below predicted EGFP-TD2.2 in right blot, Figure S3). Therefore, to maximise purity of EGFP-TD2.2 recombinant protein preparations, EGFP-TD2.2 was purified from the soluble fraction for future experiments.

### Delivery of EGFP-TD2.2 Recombinant Protein to Human Glial Cells *in vitro:* Application to Matured Glial Cells (in Isolation and Co-culture), Human Dermal Fibroblasts and Human Neural Cells

Although TD2.2 has a propensity to transduce cultures of human oligodendrocytes in vitro (Figure 2C), the selectivity of this sequence to transduce oligodendrocytes exclusively, other glial cell types and/or non-glial cells was determined. Two approaches were adopted for screening of EGFP-TD2.2 protein transduction selectivity; (i) application to cultures of mature astrocytes (other glial cell type), in isolation or in co-culture with human oligodendrocytes, and (ii) application to two representative, namely non-glial cell type (human dermal fibroblasts) and human neural cells.

To delineate cell populations in human astrocyte/human oligodendrocyte co-cultures, immature oligodendrocytes were infected with a constitutive (CMV driven)-mCherry transgene, sorted for high mCherry-expressing cells (Figure S4A), expanded in culture (Figure S4B) and matured for subsequent experiments (as for Figure S1A). To mimic the cellular environment in the brain matured human astrocytes and matured mCherry^+^ human oligodendrocytes were plated at a ratio of 5∶1 for EGFP-TD2.2 delivery experiments *in vitro*.

3 µM of purified EGFP-TD2.2 recombinant protein was applied (3×1 µM administrations over 36 hours) to cultures of (i) mature astrocytes, (ii) mature (mCherry^+^) oligodendrocytes, and (iii) mature astrocytes: mature (mCherry^+^) oligodendrocytes co-cultures (Figure 3A, C, B/D respectively). EGFP-TD2.2 was also applied to a human dermal fibroblast cell line (Figure 3E). Following EGFP-TD2.2 recombinant protein delivery and acid wash to remove peripherally bound protein, EGFP could be detected in human astrocytes (in isolation or in co-culture with mCherry^+^ human oligodendrocytes; rows A&B respectively; arrow row B) and human oligodendrocytes (in isolation or in co- culture with human astrocytes; rows C & D respectively; arrows row D). EGFP-TD2.2 could not be observed in human dermal fibroblasts by fluorescent microscopy (row E, Figure 3). These results suggested TD2.2 peptide is selective in transducing glial cells, with little ability to physically associated and/or transduce a non-glial cell line (human fibroblasts).

We adopted a more sensitive approach (flow cytometry) to ascertain comparative proportions of EGFP-TD2.2^+^ cells in all individual treatment groups. After visualization (Figure 3), relative proportions of EGFP-TD2.2^+^ cells in all individual groups were ascertained by exclusion gating of untreated cells (Figure 4A; FACS plots representative of 3 independent experiments). Flow cytometry support immunofluorescent imaging results shown in Figure 3, with 23–48% of glial cells remaining EGFP-TD2.2^+^ and minimal detection of EGFP-TD2.2^+^ human fibroblasts (raw percentage EGFP-TD2.2^+^ fibroblasts for 3 experiments; 0%, 7%, 2.8%). To further confirm the EGFP-TD2.2 recombinant protein doesn’t transduce neural cells, we repeated these experiments with neural stem cells; as observed in previous experiments, MO3.13 oligodendrocyte precursor cells actively took up the recombinant protein (41.2%) in contrast to human dermal fibroblasts, that once again, failed to transduce protein (1.3%; Figure S5). Similarly, flow cytometry analysis of EGFP-TD2.2-treated human neural stem cells further demonstrated a lack of transduction of the protein to non-neural cells (replicates 0.8% [shown], 3.3% 3.8%; Figure S5). Extending these results, we delivered EGFP-TD2.2 to immature oligodendrocytes and found EGFP-TD2.2 recombinant protein more effective at transducing immature oligodendrocytes than matured oligodendrocytes (Figure 4A & B). The transduction of human astrocytes was considerably more variable (ranging 11–47% total astrocytes, n = 3), thus not significantly different from other treatment groups.

### Kinetics of pET-EGFP-TD2.2 Recombinant Protein Transduction to Mature Human Oligodendrocytes by Sectional Confocal Microscopy

Sectional confocal microscopy was used to visualize the kinetics of EGFP-TD2.2 transduction to mCherry^+^ mature oligodendrocytes. We hypothesized mCherry expression could be used as an indication of cell volume, from which extracellular EGFP trafficking to the intracellular compartment monitored. Up to 2 hours post-application, EGFP-TD2.2 recombinant protein appears largely excluded from cells (Figure 5). Between 3–6 hours post-application, aggregates of EGFP-TD2.2 appear within the confines of mCherry expression, suggesting transduction across the plasma membrane (representative shown in arrows, Figure 5). Instead of being dispersed throughout the cytoplasmic compartment of transduced cells, EGFP-TD2.2 appears aggregated, either as free aggregate or restricted to cytoplasmic endosomes. However, it is notable that much of the aggregate EGFP-TD2.2 protein remains extracellular at this focal plane during the incubation period.

## Discussion

In the present study, we outline the theoretical formulation, and preliminary in vitro screening of a glial cell-specific, cell penetrating peptide (CPP)/transduction domain. Of the numerous CPP’s that have been described to date, generally modelled on protein/protein interactions during viral infection, few are selective in the range of cells they transduce. Applying this approach to devise a glial cell-specific targeting peptide, a survey of viruses with tropism including glial cells highlighted LCMV as an ideal candidate owing to its infection of human glia in vitro and in vivo (reviewed [36]). Although residues 169–408 of a-D are known to constitute a (LCMV) GP1 interacting motif [19], the opposing residues of GP1 have not been identified. In efforts to predict a-D binding residues of GP1, we undertook protein sequence alignments between GP1 and other known a-D binding proteins. Laminin-2 and agrin were used as representative a-D binding proteins for alignments, from which two conserved domains were identified (Figure 1B). Putative CPPs were devised from these conserved domains, a 38 amino acid domain (TD1) enriched in polar and aliphatic residues, and TD2 comprising a 29 (largely uncharged, polar) residue domain. In devising TD1 and TD2, we observed considerable identity between agrin and laminin-2; therefore, composite TD domains were constructed with cationic arginines inserted where conservation was not observed (Figure 1B).

### Initial Screening of Numerous Prospective Glial Cell-specific Targeting Peptides for Transduction to Human Oligodendrocytes

Detection of TD1.1 and TD2.1 recombinant protein in pre-washed, oligodendrocyte cultures was limited, and lost after acid washing (Figure S2B, compare top and bottom panels, left panels, TD2.1 representative of TD1.1; Note: despite being almost completely lost, one oligodendrocyte retained considerable TD2.1 protein, white arrow, Figure S2B). The loss of detectable recombinant protein following PBS/acid washes suggest detectable protein (pre-acid wash) was likely attached to the outside of treated oligodendrocytes. It also suggests the acid washing adopted for study was effective at removing peripherally-bound (TD2.2) recombinant proteins from cells (compare top and bottom panels, Figure 2C). These results suggested that the TD2.2 peptide is capable of transducing human oligodendrocytes *in vitro*. However, the ability of this protein to *selectively* transduce oligodendrocytes had yet to be determined. Therefore, to monitor the ability of TD2.2 to selectively transduce oligodendrocytes in real time, and eliminate the need for antibody labelling for detection each experiment, an EGFP-TD2.2 recombinant protein was constructed to apply to co-cultures of human astrocytes/human oligodendrocytes, and representative non-glial cell types, human dermal fibroblasts and human neural cells.

### Delivery of pET-EGFP-TD2.2 Recombinant Protein to Human Glial Cells *in vitro*

Generating a fusion protein incorporating EGFP and TD2.2 for future experiments served numerous purposes; (i) it alleviated the requirement for antibody detection of (his_6_-tagged) TD recombinant protein, enabling tracking of TD2.2 protein transduction in real-time, (ii) and is therapeutically relevant, given projected applications of the TD2.2 domain being fusion to (therapeutic) functional peptides. Repeat experiments of EGFP-TD2.2 application to human glial cells, and a representative non-glial cell type, suggested EGFP-TD2.2 is selective for glial cells, with a trend of decreasing propensity of transduction to oligodendrocytes with increasing differentiation *in vitro* (Figure 3 and 4). This opens the opportunity to deliver factors to oligodendrocyte progenitor cells to initiate maturation and remyelination (for example, [22]). Since EGFP is clearly detectable, it suggests the TD2.2 domain and linker peptide do not obstruct the secondary structure of the EGFP protein (required for fluorescence detection) to a considerable degree. Although the inability of human dermal fibroblasts and human neural cells to undertake transduction of EGFP-TD2.2 was observed in repeated experiments (Figure 3, 4, S5), suggesting glial cell-selectivity of the TD2.2 peptide, protein delivery to a suite of non-glial cell types are required *in vitro* and *in vivo* for this to be conclusively demonstrated.

### Kinetics of pET-EGFP-TD2.2 Recombinant Protein Transduction to Mature Human Oligodendrocytes by Sectional Confocal Microscopy

From 3 hours post-application, previously extracellular EGFP-TD2.2 appear to transduce the plasma membrane and appeared within the confines of cytoplasmic mCherry expression, as detected by (mid)-sectional confocal microscopy (Figure 5). However, it is notable that much of the aggregate EGFP-TD2.2 protein remains extracellular at this focal plane during the incubation period. The apparent discrepancy between this result, and considerable transduction outlined in flow cytometry results described for Figure 4, may be attributable to the sensitivity of the respective techniques ie. flow cytometry detecting cells with low concentrations of transduced protein that are below visual detection by confocal microscopy. Also, sectional microscopic images in Figure 4 represent a single focal plane of a single view of a 3-dimensional cell culture, and thus likely under-represents EGFP^+^ cells.

### Conclusion

Building on the robust results documented for non-selective transduction of Tat and Antp peptides, we modelled, constructed and screened putative oligodendrocyte targeting peptides on the viral envelope protein GP1 of LCMV, due to its semi-selective infection of human glia/oligodendrocytes *in vivo*. Of a number of putative glial-cell transduction domains described here, one domain termed TD2.2 appeared moderately effective at selectively transducing glial cells *in vitro*.

The specific targeting of cranial glia with non-immunological, therapeutic protein creates new possibilities for the treatment of MS. For example, delivery of a cleavage-resistant *Myelin Associated Glycoprotein* (MAG) to oligodendrocytes of MS patients could maintain axonal-glial cell interaction and integrity during cleavage of the endogenous form of MAG [1]. Conceptually, the protein sequence disparity between recombinant and wildtype isoforms may render the oligodendrocyte-anchored, therapeutic MAG isoform resistant to cleavage if the cleaved, circulatory form of (endogenous) MAG, termed *dMAG*, raises an autoimmune response. Alternatively, the propensity of TD2.2 to transduce oligodendrocyte precursors (Figure 4) could be exploited to initiate maturation and remyelination at sites of injury; for example, upregulation of retinoic acid (RA) signalling in oligodendrocyte precursor cell [37], elicited through (i) delivery of key RA factors, or (ii) delivery of regulatory factors for upregulation of beneficial RA molecules from endogenous loci. In addition, fusion of dominant-negative forms interferon-responsive transcription factors, or other regulatory molecules could enable mediation of oligodendrocyte immune/apoptotic responses to MS-related inflammation [38].

All aforementioned strategies could be utilized in isolation, or in concert, and may not require host immune suppression. Breaches in the blood-brain-barrier (BBB) are thought to facilitate the transmigration of immune cells to the cranial compartment in MS patients [39]; a feature that whilst detrimental to disease progression/relapse, could be exploited for (CPP)-therapeutic delivery to demyelinated lesions. However, this technology isn’t restricted to therapeutic protein delivery, or to treatment of MS. TD2.2 could be fused to RNA- or DNA-binding domains for delivery of beneficial transcripts to glia, and may also represent a method for treatment of other glial cell-related diseases, for example, genetic correction of isocitrate dehydrogenase 1 *(NADP+),* soluble (IDH1) gene mutations, common in astrocytomas and oligodendroglial tumours, with functional (i.e. non-mutant) enzyme [40]. Furthermore, cell-specific, CPP sequences could be conjugated to (PEGylated or other) nanoparticles to impart specificity [41]. Despite these possibilities, the BBB represents a significant impedance to effective delivery of CPP-fused/conjugated therapeutics to the brain for such other glial cell-related diseases.

For future therapeutic relevance to be achieved, intracellular and peripherally-bound CPP recombinant protein must not disrupt the characteristic biological processes of the transduced cell. Also, clearance of such therapeutic proteins from circulation, and function in the cytosol of transduced cells, remain biological obstacles to full and persistent therapeutic benefit to patients. To maintain persistence in circulation, functional therapeutic proteins may be fused to carrier peptide domains to protect recombinant protein from proteolysis (and promote persistence) in serum [42]. Since albumin comprises ≥50% of serum protein component, fusion of albumin-binding domains (devised from protein G of streptococcal strains) to functional moieties improves serum half-life 4–5 fold [42]. Similarly, frameshifts caused by single base pair deletions to the C-terminus of the bacteriophage DNA binding protein P22 Arc, extending the reading frame past the natural stop codon by 25 residues (^51-^IGRKVEAPTAVTVRASVVSKSLEKNQHE^-78^), conferred protection against intracellular proteolysis [43]. Fusion of this domain to an unrelated protein caused 4-fold improvements in its half-life.

Random and unpredictable interactions between functional domains and CPP/accessory domains within the same fusion protein can potentially impair or abrogate function of translocated fusion protein. Design of an intracellular protease cleavage motif into linker sequences (ie. protein sequence interceding CPP and functional domains) liberates the functional peptide from redundant accessory sequences upon translocation to the cytosol and cleavage. To illustrate, *in situ* recognition and cleavage of a 12 residue Furin serine protease cleavage motif (-TRHRQPRGWEQL-), designed between 2 functional domains in fusion proteins, results in fold-increases in activity upon cleavage [44]. A number of reports describe recombinant proteins remaining restricted to cytosolic endosomes following endocytosis [31]. Use of endosmolytic agents (eg. influenza hemagglutinin-derived peptides) are effective at prompting rupture of endosomes *in vitro*. However, issues about toxicity limit their use in a therapeutic setting. Also, amino acids of human protein are exclusively of L-stereoisomer conformation (with D-stereoisomer residues only found in some bacterial peptides or some antibiotics). Therefore, evolutionary selection for proteins comprising L-stereoisomer residues concomitantly selected for proteolytic enzymes that preferentially cleave L-amino acid substrates. Incorporation of D-stereoisomer residues at proteolytic cleavage sites of recombinant proteins reduces the substrate/enzyme recognition and subsequent cleavage.

## Supporting Information

Figure S1 **Assessment of the MO3.13 Oligodendrocyte, 4-β-phorbol 12-myristate Maturation Protocol by Morphology and RT-PCR screen.** (A) Morphological changes to MO3.13 precursor cell line following 4-**β**-phorbol 12-myristate treatment; from rounded morphology to characteristic spindle-shaped morphology of mature oligodendrocyte [28]. (B) Confirmation of endogenous expression of a-Dystroglycan in human oligodendrocytes, and absence in astrocytes. (C) Mature oligodendrocyte markers 2′,3′-cyclic nucleotide 3′-phosphodiesterase (CNPase) and Myelin Basic Protein (MBP) are upregulated in the MO3.13 oligodendrocyte cell line upon application of the PMA maturation protocol [30].
(TIFF)

Figure S2 **Construction of TD protein expression vectors, and preliminary TD delivery experiment to human oligodendrocytes.** (A) Schematic of construction of protein expression vector from PCR/restriction digest products. (B) Immunofluorescence images of oligodendrocyte cultures treated with TD2.1 (representative of TD1.1 and EGFP-TD1.1), and TD2.2 recombinant proteins. Cells were washed with 0.2 M Glycine-HCl to remove peripherally-bound, non-transduced recombinant protein [31].
(TIF)

Figure S3 **Construction of the pET28c-EGFP-TD2.2 Protein Expression Vector.** Schematic of construction of the pET28c-EGFP-TD2.2 protein expression vector. Agarose gels showing DNA inserts for ligation to expression vector. Western Blot of expressed TD recombinant proteins purified under soluble (left blot) or insoluble (denaturing) conditions (right blot). Predicted proteins are indicated to right of each gel.
(TIF)

Figure S4 **Retroviral transduction and cell sorting of human oligodendrocytes with constitutive (CMV)-mCherry transgene.** A) MO3.13 human oligodendrocytes (immature) were infected with a constitutive (CMV driven)-mCherry transgene and flow cytometry undertaken to enrich for mCherry^high^ cells (representing top 12% of mCherry expressing cells; sort population indicted). Flow cytometry plots show uninfected cells (left hand side) and sorted mCherry+ cells (gated; right hand side). (B) Phase contrast and Immunofluorescent images (left and right hand side, respectively) of sorted mCherry^+^ oligodendrocytes undergoing expansion *in vitro*.
(TIF)

Figure S5 **Flow Cytometry Analysis of Relative Proportions of Immature Oligodendrocytes, Dermal Fibroblasts and Human Neural Cells to undertake EGFP-TD2.2 Transduction.** From left, flow Cytometry plots show (i) untreated human neural cells, and (1 µM) EGFP-TD2.2-treated (ii) human human neural cells, (iii) MO3.13 oligodendrocyte precursor cells (positive control), and (iv) human dermal fibroblasts (negative control). Representative plots of 3 replicates; percentages indicate EGFP+ gated events.
(TIF)
