# Supplementary figures and images for: Design and Screening of a Glial Cell-Specific, Cell Penetrating Peptide for Therapeutic Applications in Multiple Sclerosis
Source: PLoS One. 2012 Sep 25;7(9):e45501. doi: 10.1371/journal.pone.0045501 (PMC3458069; doi:10.1371/journal.pone.0045501)

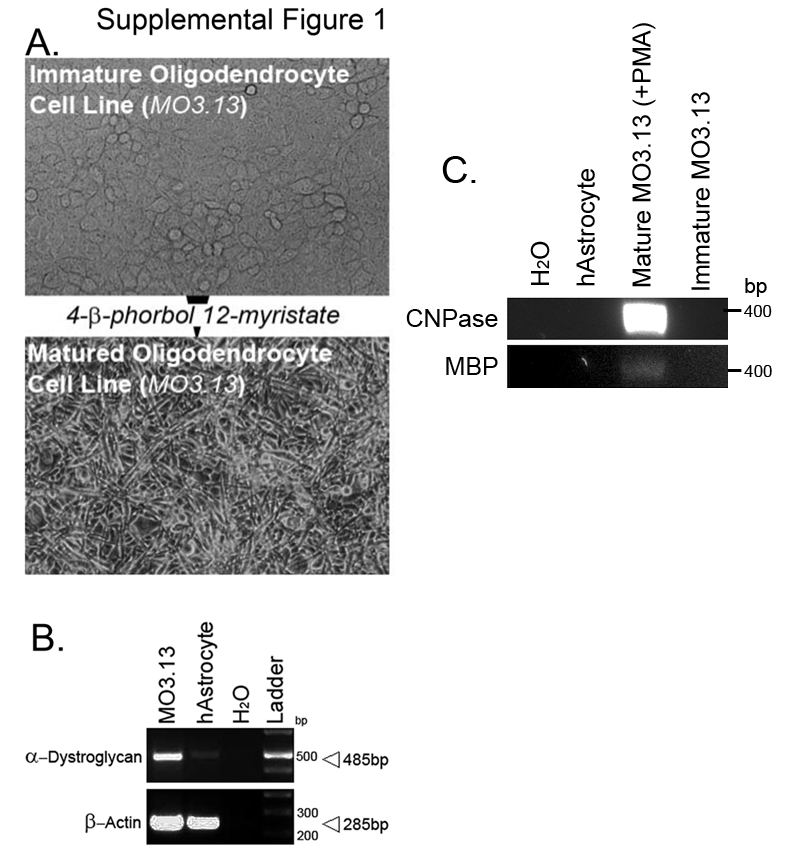

Supplement: Figure S1 — Assessment of the MO3.13 Oligodendrocyte, 4-β-phorbol 12-myristate Maturation Protocol by Morphology and RT-PCR screen. (A) Morphological changes to MO3.13 precursor cell line following 4-β-phorbol 12-myristate treatment; from rounded morphology to characteristic spindle-shaped morphology of mature oligodendrocyte [28]. (B) Confirmation of endogenous expression of a-Dystroglycan in human oligodendrocytes, and absence in astrocytes. (C) Mature oligodendrocyte markers 2′,3′-cyclic nucleotide 3′-phosphodiesterase (CNPase) and Myelin Basic Protein (MBP) are upregulated in the MO3.13 oligodendrocyte cell line upon application of the PMA maturation protocol [30]. (TIFF) [file pone.0045501.s001.tiff]

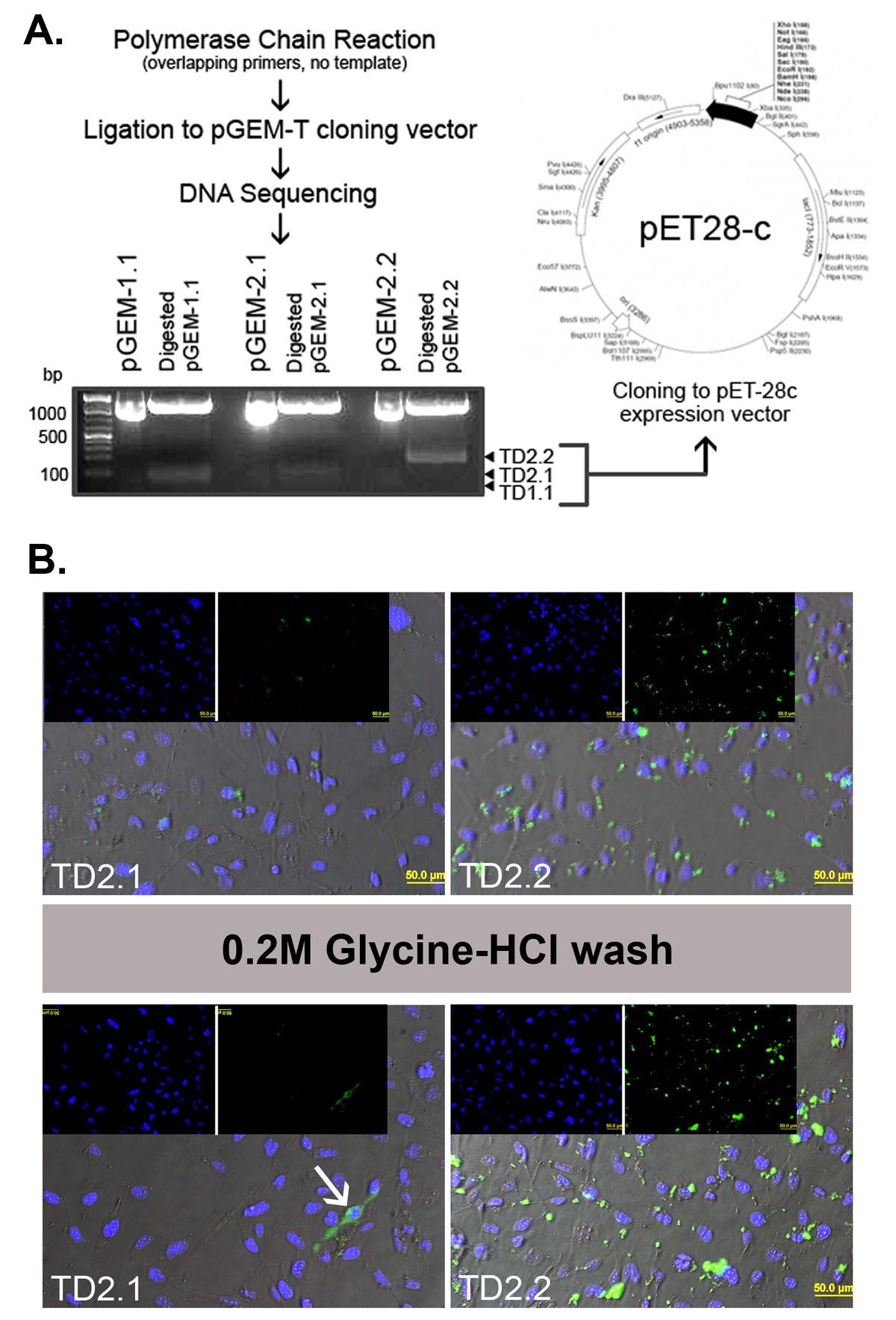

Supplement: Figure S2 — Construction of TD protein expression vectors, and preliminary TD delivery experiment to human oligodendrocytes. (A) Schematic of construction of protein expression vector from PCR/restriction digest products. (B) Immunofluorescence images of oligodendrocyte cultures treated with TD2.1 (representative of TD1.1 and EGFP-TD1.1), and TD2.2 recombinant proteins. Cells were washed with 0.2 M Glycine-HCl to remove peripherally-bound, non-transduced recombinant protein [31]. (TIF) [file pone.0045501.s002.tif]

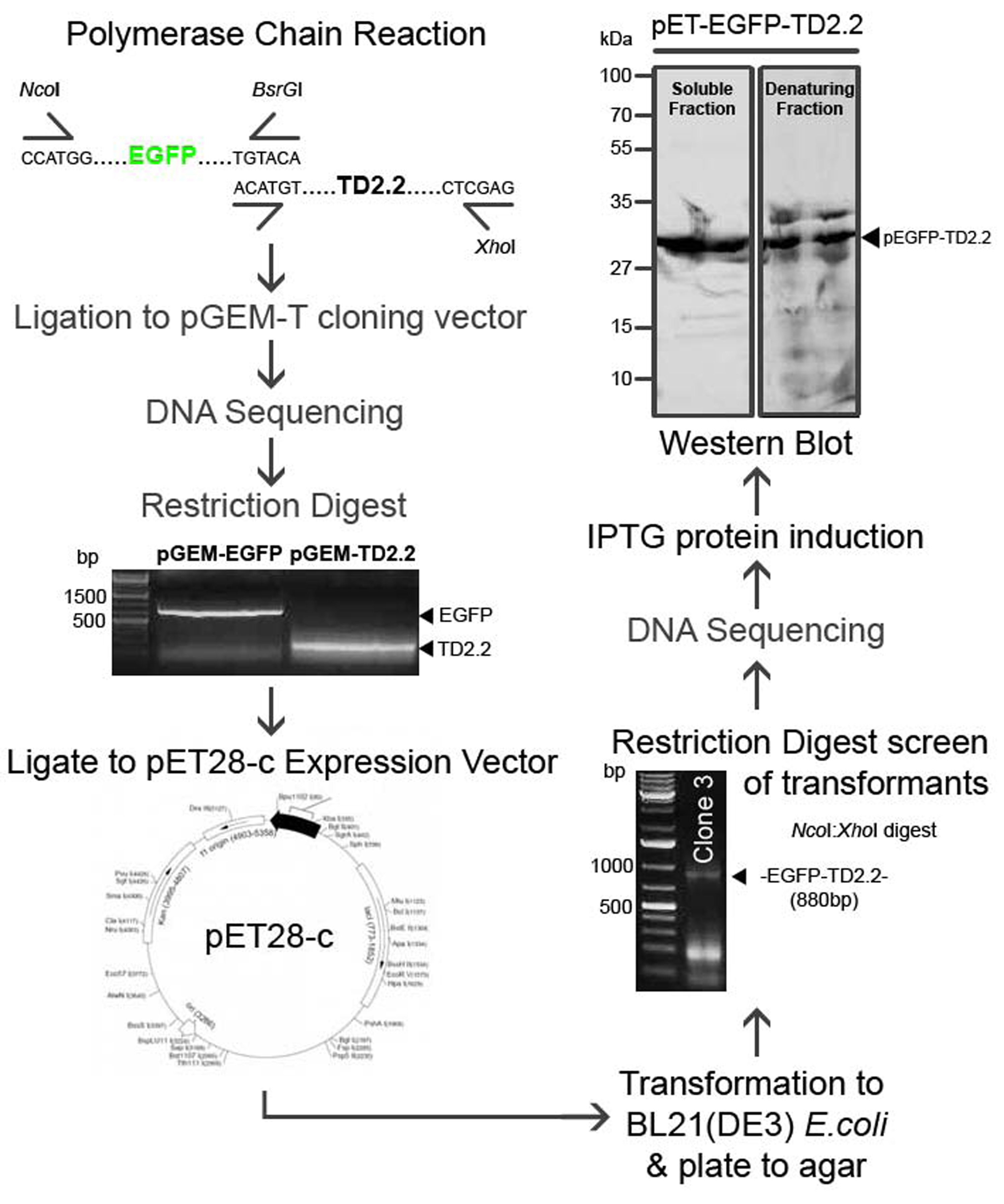

Supplement: Figure S3 — Construction of the pET28c-EGFP-TD2.2 Protein Expression Vector. Schematic of construction of the pET28c-EGFP-TD2.2 protein expression vector. Agarose gels showing DNA inserts for ligation to expression vector. Western Blot of expressed TD recombinant proteins purified under soluble (left blot) or insoluble (denaturing) conditions (right blot). Predicted proteins are indicated to right of each gel. (TIF) [file pone.0045501.s003.tif]

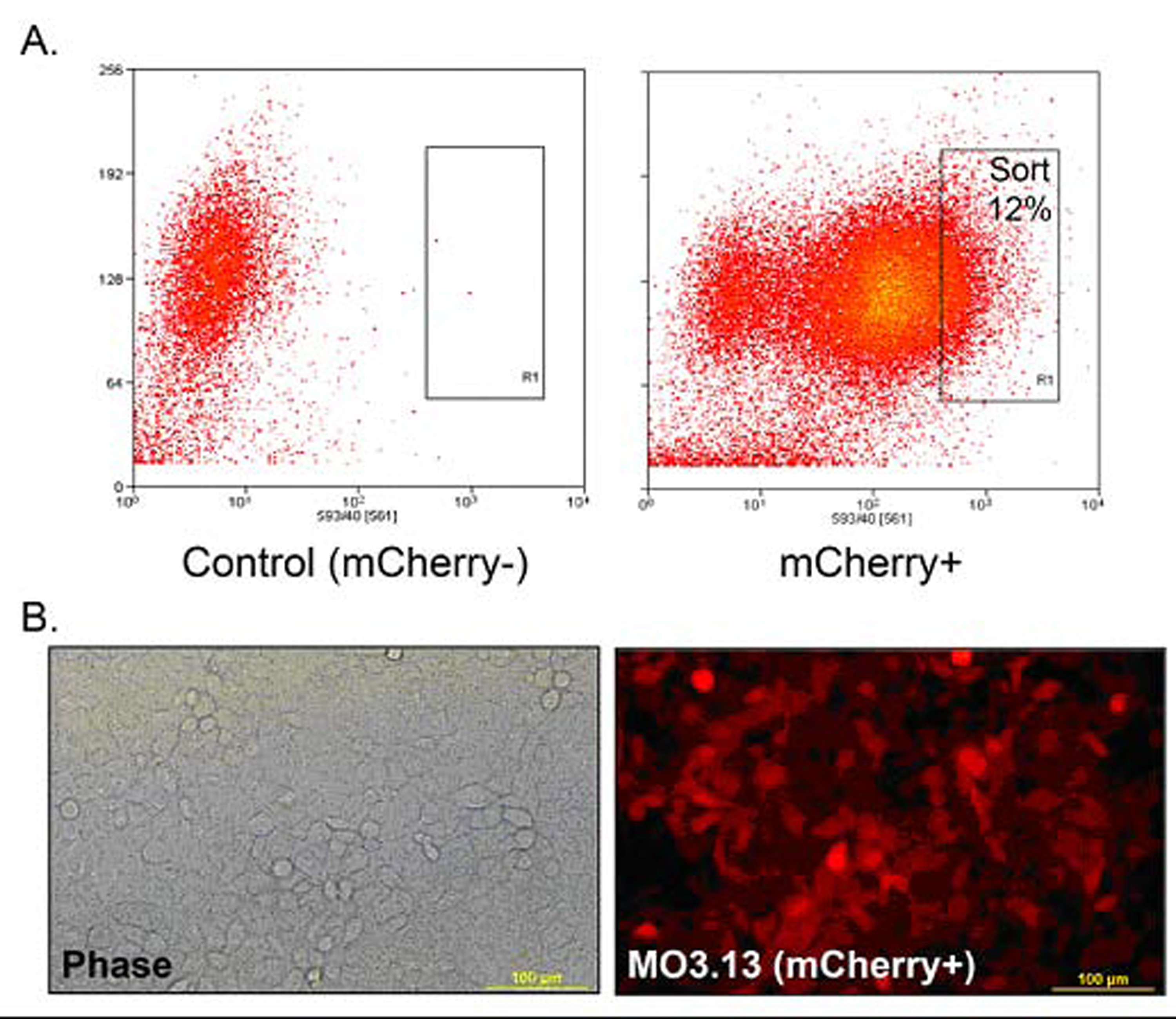

Supplement: Figure S4 — Retroviral transduction and cell sorting of human oligodendrocytes with constitutive (CMV)-mCherry transgene. A) MO3.13 human oligodendrocytes (immature) were infected with a constitutive (CMV driven)-mCherry transgene and flow cytometry undertaken to enrich for mCherryhigh cells (representing top 12% of mCherry expressing cells; sort population indicted). Flow cytometry plots show uninfected cells (left hand side) and sorted mCherry+ cells (gated; right hand side). (B) Phase contrast and Immunofluorescent images (left and right hand side, respectively) of sorted mCherry+ oligodendrocytes undergoing expansion in vitro. (TIF) [file pone.0045501.s004.tif]

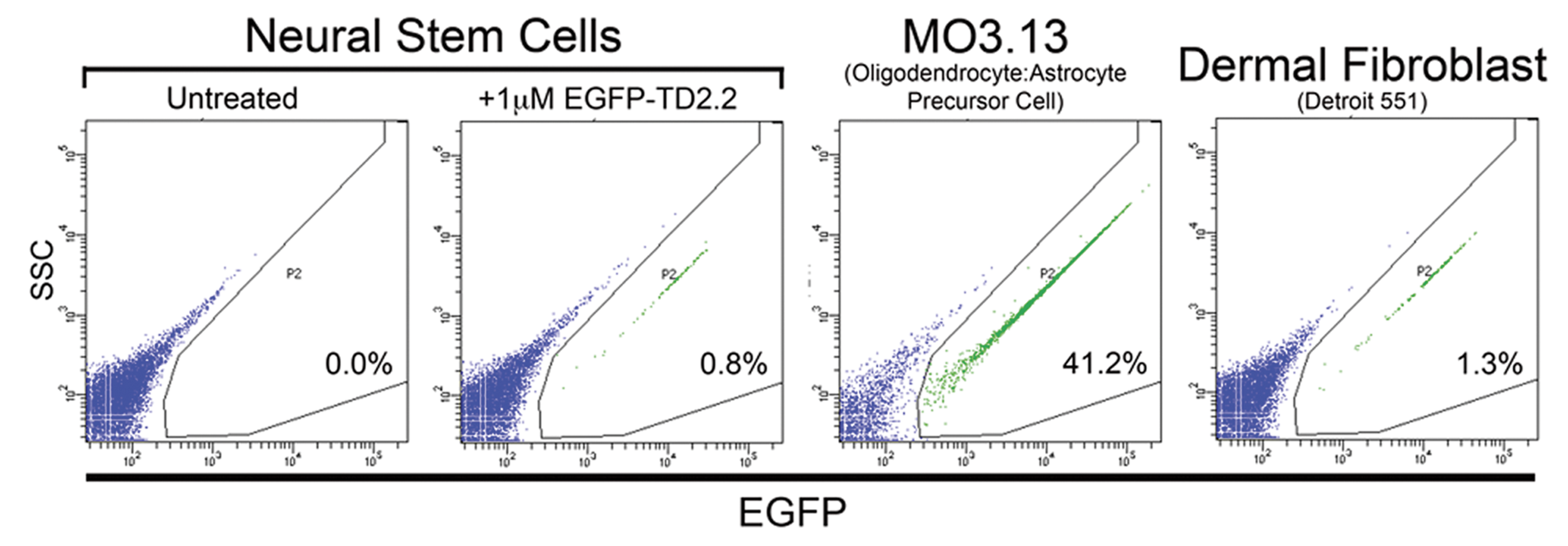

Supplement: Figure S5 — Flow Cytometry Analysis of Relative Proportions of Immature Oligodendrocytes, Dermal Fibroblasts and Human Neural Cells to undertake EGFP-TD2.2 Transduction. From left, flow Cytometry plots show (i) untreated human neural cells, and (1 µM) EGFP-TD2.2-treated (ii) human human neural cells, (iii) MO3.13 oligodendrocyte precursor cells (positive control), and (iv) human dermal fibroblasts (negative control). Representative plots of 3 replicates; percentages indicate EGFP+ gated events. (TIF) [file pone.0045501.s005.tif]
